# Current Understanding and Recent Developments in Common Variable Immunodeficiency Associated Autoimmunity

**DOI:** 10.3389/fimmu.2019.02753

**Published:** 2019-12-10

**Authors:** Jessica D. Gereige, Paul J. Maglione

**Affiliations:** Department of Pulmonary, Allergy, Sleep & Critical Care Medicine, Boston Medical Center, Boston University School of Medicine, Boston, MA, United States

**Keywords:** primary immunodeficieny, common variable immunodeficiency, autoimmunity, cytopenia, genetics, microbiome, precision therapy

## Abstract

Common variable immunodeficiency (CVID) is the most prevalent symptomatic primary immunodeficiency and comprises a group of disorders with similar antibody deficiency but a myriad of different etiologies, most of which remain undefined. The variable aspect of CVID refers to the approximately half of patients who develop non-infectious complications in addition to heightened susceptibility to infection. The pathogenesis of these complications is poorly understood and somewhat counterintuitive because these patients that are defined by their immune futility simultaneously have elevated propensity for autoimmune disease. There are numerous aspects of immune dysregulation associated with autoimmunity in CVID that have only begun to be studied. These findings include elevations of T helper type 1 and follicular helper T cells and B cells expressing low levels of CD21 as well as reciprocal decreases in regulatory T cells and isotype-switched memory B cells. Recently, advances in genomics have furthered our understanding of the fundamental biology underlying autoimmunity in CVID and led to precision therapeutic approaches. However, these genetic etiologies are also associated with clinical heterogeneity and incomplete penetrance, highlighting the fact that continued research efforts remain necessary to optimize treatment. Additional factors, such as commensal microbial dysbiosis, remain to be better elucidated. Thus, while recent advances in our understanding of CVID-associated autoimmunity have been exciting and substantial, these current scientific advances must now serve as building blocks for the next stages of discovery.

## Introduction

The diagnosis common variable immunodeficiency (CVID) is used to denote a group of disorders that, together, account for more than 50% of symptomatic primary immune deficiencies (PID) (1) with an estimated incidence of 1:50,000–1:25,000 (2). The term common variable immunodeficiency was originally used to describe patients with a primary antibody deficiency who did not meet criteria for the more well-defined PIDs such as Burton's agammaglobulinemia (3). We have come to appreciate that CVID is best understood to constitute a group of hypogammaglobulinemic disorders with heterogeneous phenotypic presentations, rather than a single entity (4). The application of genomics has only furthered the evidence of heterogeneity within CVID.

Diagnostic criteria for CVID have evolved since it was first made a diagnosis of exclusion in 1971 by the World Health Organization. The International Consensus Document on CVID put forward in 2016 agreed on a definition requiring IgG levels two standard deviations below the age-appropriate reference as well as either low IgA or IgM levels, and poor antibody response to vaccination in an individual that is at least 4 years old with no secondary cause of hypogammaglobulinemia (1). The European Society of Immune Deficiencies (ESID) diagnostic criteria differ slightly from these criteria in that they require the presence of symptoms such as infections or autoimmune manifestations, in addition to the laboratory abnormalities cited above, to make the diagnosis of CVID (5). Notably, non-infectious presentations are an under-recognized feature of CVID and often the predominant clinical presentation, resulting in diagnostic delays of several years in many cases (6). While historically the clinical presentation of CVID was focused on the susceptibility to infections, the decision to include autoimmune and inflammatory conditions as primary clinical presentations reflects the heterogeneity of CVID and highlights the importance of recognizing these non-infectious entities as a feature of this immune deficiency.

Immunoglobulin replacement therapy is the standard of care for CVID. Since the widespread adoption of this treatment, mortality of patients with CVID has decreased from 30% in the early 1990s (7) to 15% in the early 2000s (8), in a cohort of 240 patients in the United Kingdom and 334 patients from the ESID registry, respectively, all followed for approximately two decades. The improved survival in CVID patients has been attributed to the reduction of infectious complications thanks to the widespread use of immunoglobulin replacement and improved anti-microbial therapies (9–12). While overall survival has improved, patients with CVID continue to have reduced survival compared to age-matched controls (13). When comparing mortality within CVID patients, subjects with at least one non-infectious complication had significantly higher mortality compared to patients with only infectious complications (13, 14). These non-infectious complications including autoimmune, gastrointestinal, pulmonary, lymphoproliferative, and malignant complications (1), are not ameliorated by immunoglobulin replacement therapy alone. The clear necessity to address non-infectious complications of CVID has led to attempts at categorizing these heterogeneous disorders into distinct phenotypes, with the hopes of elucidating endotypes. The ultimate goal of such stratification of CVID is to identify targeted treatments that will improve outcomes in CVID patients with non-infectious complications (14–16).

The first attempt at phenotyping CVID patients was done by Chapel et al. (14) by categorizing the various associated complications and looking at the independence of one from the other. They identified five distinct phenotypes: no complications, autoimmunity, polyclonal lymphocytic infiltration, enteropathy, and lymphoid malignancy (14). Studies that followed have confirmed categorization of CVID based upon the presence of complications, with certain features like autoimmunity, lymphocytic interstitial lung disease, and lymphoid hyperplasia typically occurring together (6, 17). Assuming that CVID endotypes present within these phenotypic clusters, the pathogenesis and genetic mechanisms underlying the disease may be related. Autoimmunity and immune deficiency have been shown to have genetic overlap and occur together beyond CVID (18–20), suggesting a common pathophysiologic mechanism underlying both forms of immune dysregulation. In this review, we focus on one aspect of immune dysregulation, the autoimmune manifestations of CVID, providing an overview of these complications as well as an update on research and treatment advances.

## Overview of Autoimmune Disease in Common Variable Immunodeficiency

The initial clustering of CVID patients with autoimmunity included organ specific autoimmune disease (e.g., Grave's thyroiditis, insulin dependent diabetes mellitus), systemic autoimmune disease (e.g., rheumatoid arthritis, systemic lupus erythematosus), and autoimmune cytopenias (e.g., immune thrombocytopenia, autoimmune hemolytic anemia) (14). Further analysis on two other cohorts showed that within the autoimmune cluster, only autoimmune cytopenias had decreased survival and that organ-specific and systemic autoimmune disease showed no association with cytopenias or the other clinical phenotypes (16). This led to a revision of the clinical phenotypes with more emphasis being placed on autoimmune cytopenias as opposed to autoimmunity in general (16). Unbiased network clustering in a separate CVID cohort yielded similar phenotypes, with systemic and organ-specific autoimmune diseases clustering separately from autoimmune cytopenias (21). While this distinction likely carries implications regarding underlying pathophysiology, many studies continue to combine autoimmune cytopenias with other autoimmunity as they compare CVID patients. For this reason, we will make distinctions between autoimmune cytopenias and organ specific or systemic autoimmunity in this review.

Autoimmune diseases are one of the most common non-infectious complications, occurring in ~20–30% of patients with CVID, with autoimmune cytopenias being the most common (9, 22, 23). In a European cohort of 2700 CVID patients taken from the ESID registry, autoimmune cytopenias were found to be 700 times more prevalent in CVID patients compared to the general population (9). Among autoimmune cytopenias, autoimmune thrombocytopenia and autoimmune hemolytic anemia occur most frequently, either separately or concurrently as Evan's syndrome. Autoimmune neutropenia also occurs in CVID, although more rarely than thrombocytopenia or anemia (9, 24). Importantly, the diagnosis of cytopenia precedes that of CVID by several years in up to 60% of patients (22).

Autoimmune cytopenias are often associated with other non-infectious complications in CVID. Compared with other CVID patients, those with ILD are more likely to have had autoimmune cytopenias (6, 25). Conversely, CVID patients with autoimmune cytopenias had a higher frequency of CVID-associated non-infectious complications, including granulomatous and lymphoproliferative disease, as well as organ-specific autoimmune disease, but interestingly, not systemic autoimmunity (26). In a recent study of a 295 patient CVID cohort in the Czech Republic, immune thrombocytopenic purpura (ITP) was identified as a risk factor for malignancy, with over a 3-fold increase compared to those without ITP (27). Splenomegaly was also more common in patients with autoimmune cytopenias and was found to share some immunophenotypic characteristics, but the pathophysiology of this link is still not clearly understood (28).

Though autoimmune cytopenias are highly associated with CVID, other forms of autoimmunity have also been frequently reported. In a recent study of 870 CVID patients from the USIDNET registry, 5% were found to have rheumatologic disease, which accounted for 40% of the autoimmune complications of this cohort. Although the male-to-female ratio was almost equal for overall autoimmune complications, there was a clear female predominance for the rheumatologic manifestations (24). Several other studies have shown a similar female predominance for systemic autoimmune complications in CVID (29, 30). The most common rheumatologic manifestation reported in CVID is inflammatory arthritis (juvenile and adult), occurring in ~3% of patients (13, 24, 31, 32). Other rheumatologic manifestations include systemic lupus erythematosus, Sjogren's disease, Behcet's disease, and psoriasis (24, 32). Among organ specific autoimmune manifestations, hypothyroidism was the most prevalent at 3.5%, followed by alopecia areata and vitiligo at 2.7%, and type 1 diabetes at 1.6% in an ESID registry of 2700 CVID patients (9). Autoimmunity may also underlie gastrointestinal complications of CVID, including inflammatory bowel disease, autoimmune enteropathy, and autoimmune gastritis (33).

## Immunophenotypic Markers Associated With Autoimmunity in CVID and Implications on Pathogenesis

### T Cell Dysregulation in CVID Autoimmune Disease

While PIDs in general are associated with a higher risk of autoimmune manifestations, when comparing across all PIDs, the greatest risk of autoimmune cytopenia and rheumatologic disease was seen in two specific subsets of PID: CVID and PID with T-cell deficiencies (34). This finding highlights the importance of T cell dysfunction in CVID autoimmunity and demonstrates that pathogenesis of this immune disorder extends beyond defects of B cells exclusively. Indeed, CVID patients with autoimmunity have been found to have lower total T cells compared to those without autoimmunity (30). Such T cell deficiency is not necessarily profound, as a CD4+ T cell count <200 or significantly impaired lymphocyte proliferation may result in categorization as a combined immunodeficiency rather than CVID (35). When Chapel et al. classified CVID patients into specific phenotypes, they found that low proportions of CD8+ T cells were predictive of autoimmunity; in fact, in this cohort, each additional 10% increase in CD8+ T cells reduced the odds of autoimmunity by 18% (14). Later studies found that autoimmunity in CVID is associated with lower naïve CD8+ T cells specifically, with terminally differentiated CD8+ T cells actually increased, suggesting a hyperactivated T cell phenotype as the defining feature (36). While several studies have shown reduced CD4+ cells in CVID (29, 37), those with autoimmune cytopenias and organ specific autoimmunity had the most significantly reduced CD4+ T cells (36, 38, 39). Within CD4+ T cells, autoimmunity in CVID was particularly associated with reduced number of regulatory T cells (T_R_) compared to CVID patients without autoimmunity (39, 40). Expression of the canonical T_R_ transcription factor forkhead box P3 (FOXP3) was reduced in CVID patients with autoimmunity compared to those without (39), and the suppressive activity of T_R_ cells in CVID with autoimmunity was also reduced, with the degree of dysfunction correlating with the extent of FOXP3 downregulation (41). Other findings in CVID patients with autoimmunity include lower CCR7+ T cells, also considered to be key mediators in immune tolerance (42). Overall, T cell-mediated processes that help promote immune tolerance appear to break down in a subset of CVID patients, contributing to the development of autoimmunity.

In addition to loss of naïve and regulatory T cells, an increase in T helper type 1 (T_H1_) and T follicular helper (T_FH_) CD4+ T cells have been described in association with autoimmunity in CVID. T_FH_ cells provide help to activate and diversify B cell responses within secondary and tertiary lymphoid tissues (43). T_FH_ are elevated in CVID patients with autoimmunity, particularly those producing type 1 cytokines or otherwise known as T_FH1_ (44). While T_FH_ provide most of their function within germinal centers, it is notable that their increase is associated with germinal center enlargement and disorganization in CVID patients with autoimmunity (45). This increased T_FH_ development has been linked with greater IgA deficiency and resultant endotoxemia, presumably due to bacterial translocation from mucosal surfaces in the absence of IgA (46). Expansion of T_H17_ cells has also been associated with autoimmunity in patients with CVID (47, 48). Thus, it is clear that T cell dysregulation, particularly loss of regulatory subsets with concurrent increase in proinflammatory lymphocytes, is a fundamental feature of CVID patients with autoimmunity. Continued efforts toward improved understanding of this form of immune dysregulation will be vital to improving treatment of CVID associated autoimmune disease.

### B Cell Dysfunction in CVID Autoimmune Disease

Low frequency of T_R_ cells in CVID patients with autoimmunity is associated with expansion of a particular B cell type, CD21^low^ B cell (37), linking T and B cell pathology in CVID. Early studies have shown that reduced switched memory B cell (CD19+CD27+IgD–) percentage correlates more strongly with autoimmunity in patients with CVID compared to serum IgG levels (49). Indeed, patients with reduced numbers of switched memory B cells (≤0.55% of B-cells) had greater than a 3-fold increase in their risk of autoimmune cytopenias and systemic autoimmune disease (50). Subsequent studies found increased proportions of CD21^low^ B cells in patients with CVID (37, 51, 52), with clustering of these low levels in patients with autoimmunity (28, 53). Interestingly, non-CVID patients with autoimmune disease have also been found to have expansion of CD21^low^ B cells (54). These cells were found to have preferential homing to peripheral tissues such as the synovial fluid of rheumatoid arthritis patients and the bronchoalveolar space of CVID patients (55). Further analysis found that CD21^low^ B cells from both rheumatoid arthritis and CVID patients expressed germline autoreactive antibodies which recognized nuclear and cytoplasmic structures (56). Authors concluded that CD21^low^ B cells are a “distinct, polyclonal, pre-activated, partially autoreactive, functionally attenuated B cell population with preferential enrichment in peripheral tissues” thus offering another possible mechanism of autoimmunity in patients with CVID (55).

Notably, CD21^low^ B cells were found to produce significantly more IgM than naïve B cells after stimulation with CD40L, IL-2, and IL-10 (55). Along these lines, CVID patients with autoimmunity have been found to have higher levels of IgM compared with non-autoimmune phenotypes (31, 57). High levels of IgM have also been associated with autoimmunity in other PIDs, such as Wiskott-Aldrich syndrome (58). While this may be a marker for increased risk of autoimmune disease, and may be related to the aforementioned CD21^low^ B cells, there may be a pathogenic role for IgM autoantibodies, as IgM may underlie the autoimmune cytopenias that are the predominant autoimmune manifestation of CVID (57).

B cell receptor diversity is diminished in CVID patients with autoimmunity (59). As a consequence of this diminished B cell repertoire, the presence of certain B cell receptors with autoreactive proclivity, such as those that express the VH4-34 heavy chain, may be more prominent in CVID patients as has been demonstrated in other forms of PID associated with autoimmunity (60, 61). Thus, B cell developmental defects that impair immunity may also contribute to the propensity for autoimmunity in some CVID patients.

B cell-activating factor (BAFF), a cytokine that promotes both maturation and survival of B cells, has long been linked with autoimmunity when its levels are elevated (62–64). BAFF also stimulates T cells via the BAFF receptor (BAFF-R) and skews the inflammatory response by augmenting T_H1_ cytokine production (65). Though BAFF levels were found to be elevated in CVID, an association with autoimmunity was not found (66). One possible explanation for this discrepancy is that the sample size of this cohort did not provide enough power to reach significance (only 17 of 77 patients had autoimmunity). In a separate study, BAFF levels were elevated in CVID patients with active interstitial lung disease, an inflammatory pulmonary disease linked with autoimmune cytopenias (17, 25). On the other hand, BAFF levels were not elevated in those with quiescent stable disease, suggesting that increases of this cytokine might be closely tied to disease activity (67) thus offering another possible explanation for the lack of association in the aforementioned cohort. While shedding of BAFF receptors has been postulated to regulate BAFF-driven inflammation, there was no relationship of serum levels of the BAFF receptor B cell maturation antigen (BCMA) and the development of autoimmunity in CVID (68, 69). Yet, elevated BAFF levels have been shown to inhibit negative selection of autoreactive B cells, in CVID autoimmunity as in other diseases, which apparently contributes to the increased autoimmunity seen in CVID patients with mutations in the BAFF receptor transmembrane activator and calcium-modulator and cyclophilin ligand interactor (TACI) (70–72). Further studies are needed to refine our understanding of the complex relationship between BAFF and autoimmunity in CVID.

**Figure 1:**
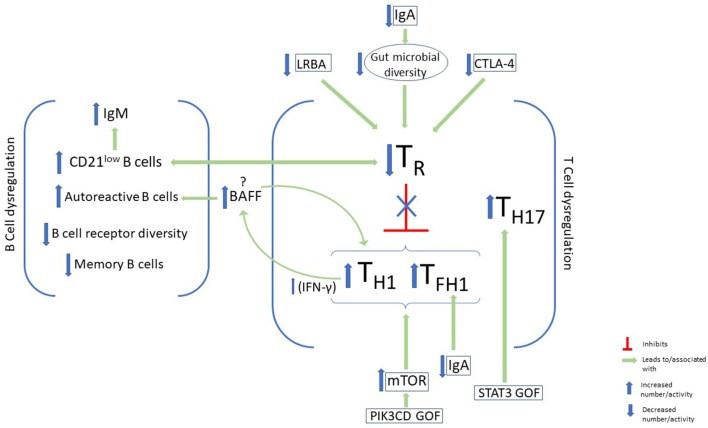
B-cell and T-cell dysregulation and immunophenotypic characteristics of autoimmunity in CVID. Some features of B-cell dysregulation in CVID patients with autoimmunity include elevated CD21^low^ B cells and IgM and increased autoreactive B cells in the periphery. On the other hand, memory B-cells, and B-cell receptor diversity are decreased. Disturbance of T-cell homeostasis, with a decrease in T regulatory cells (T_R_) and a skew toward type 1 immunity, has been associated with autoimmunity in CVID. Factors that have been identified in CVID patients which lead to downregulation of T_R_ cells include LRBA and CTLA-4 deficiency, as well as IgA deficiency. Upregulation of mTOR activity in GOF PIK3CD may also promote this immune dysregulation, while STAT3 GOF leads to expansion of T_H17._ BAFF augments T_H1_ cytokine production via its effects on the BAFF receptor on T cells, and promotes the survival of autoreactive antibodies via its receptor on B cells.

Other biomarkers of immune dysregulation have been linked with autoimmunity, as well as other non-infectious complications, in CVID. Importantly, an elevated T_H1_ signature has been found in the peripheral blood of these patients (73). This signature includes the prototypical T_H1_ cytokine interferon-γ (IFN-γ) and its downstream effects. This heightened T_H1_ signature in the blood is consistent with the heighted T_H1_ cellular response previously mentioned to be increased in CVID patients with autoimmune complications and elevated CD21^low^ B cells. Elevated IFN-γ producing innate lymphoid cells have also been found to distinguish CVID patients with non-infectious complications (74). Thus, systemic immune dysregulation favoring T_H1_ cytokine production appears to be an important feature of CVID patients with non-infectious complications, yet the pathological basis of this skewed cytokine response remains unclear. Likewise, the therapeutic benefit of trying to neutralize this heightened T_H1_ response remains inadequately explored (Figure 1).

## Genetics of CVID Autoimmunity

As CVID is a phenotypically heterogeneous disease, the expansive genetic landscape of these patients is perhaps unsurprising. While many CVID patients may have polygenic disease, recent advances in next generation sequencing (NGS) techniques have increased the discovery of monogenic forms of CVID to 15–30% of cases (75, 76) from 2–10% in 2016 (77). The majority of identified monogenic mutations encode proteins present in immune cells, which may reflect the nature of this immune disorder or a bias of the genomic analysis that is still nascent in its sophistication (77, 78). Out of the 12 monogenic mutations listed on the Online Mendelian Inheritance in Man (OMIM) database (79) in association with CVID, we will focus on those associated with autoimmunity. It is worth noting that while these mutations are associated with a clinical presentation that fits the diagnosis of CVID, many instances may not meet the full diagnostic criteria, in particular the extent of hypogammaglobulinemia typically needed. As these mutations were described in patients with CVID or CVID-like disorders and are likely to be encountered in a clinical evaluation of such patients, we include them in our discussion of CVID-associated autoimmunity even though there is an emerging trend to categorize them separately.

Defects in the TACI, a BAFF and APRIL (a proliferation inducing ligand) receptor encoded by the *TNFRSF13B* gene, is one of the first mutations to be linked to CVID (80). It is also among the most common genetic variants found, detected in up to 10% of CVID patients who can be either heterozygous or homozygous for the mutation (81). Heterozygous TACI mutations may be more appropriately defined as a risk factor for CVID, as some are not adequately rare to be considered monogenic etiologies and are frequently found in unaffected individuals (81). Notably, CVID patients heterozygous for the *TNFRSF13B* variant have a higher risk of developing autoantibody-mediated autoimmunity than those with homozygous mutations (82). It has been hypothesized that this difference may be due to the level of dysfunction in the TACI receptor: by regulating the function of several other receptors, TACI may be involved in central B cell tolerance and that reduced function results in loss of tolerance and resultant autoimmunity. By contrast, in homozygous individuals, the complete loss of TACI function results in the inability to maintain continuous autoantibody production that would otherwise result in autoimmunity (82).

LRBA (lipopolysaccharide-responsive beige-like anchor) and CTLA-4 (cytotoxic T-lymphocyte-associated protein 4) deficiencies are two closely related protein deficiencies that were detected in patients with CVID and autoimmunity (83). While mutations in *LRBA* and *CTLA4* have phenotypic variance thought to be due to incomplete penetrance and epigenetic changes, a common finding in these patients is hypogammaglobulinemia and early onset severe autoimmunity (77). CTLA-4 is an inhibitory T cell receptor that negatively regulates immunity by inhibiting excessive T cell activation and maintaining immune tolerance via its effect on T_R_ cells (83). LRBA, on the other hand, is thought to play a role in CTLA-4 cell surface expression, hence the phenotypic similarities in the two deficiencies (84). Deficiencies in both these proteins thus cause excessive T cell activation and breakdown of immune tolerance, resulting in autoimmunity. They are both examples of how T cell-intrinsic genetic defects can lead to hypogammaglobulinemia, further highlighting how T cell dysfunction is key to the pathogenesis of at least some cases of CVID.

Gain-of-function (GOF) mutations in *STAT3* have been identified in CVID as well as those with less profound antibody defects (75, 78). Patients with STAT3 GOF mutations also present with early-onset and quite severe manifestations of autoimmune disease (85, 86). One mechanism through which STAT3 is thought to lead to autoimmunity is by promoting the activation and expansion of autoimmunity-associated T_H17_ cells (47, 48). While a heightened T_H1_ response has been linked to CVID complications, features of these STAT3 GOF patients indicate that other forms of hyperactivated T cell responses, namely T_H17_, may also promote an autoimmune CVID phenotype. Additionally, increased STAT3 activation may impair B cell differentiation (87) leading to hypogammaglobulinemia and heightened autoreactivity found in association with CVID or more mild forms of hypogammaglobulinemia. Thus, STAT3 GOF may have both B cell-extrinsic and -intrinsic effects contributing to the immunological phenotype of affected patients.

Class IA phosphoinositide 3-kinases (PI3Ks) are heterodimeric lipid kinases that are involved in regulating cell growth, survival, and activity. Recently, a GOF mutation in the gene *PIK3CD* encoding PI3Kδ has been found in patients with CVID-like disease and autoimmunity. PI3Kδ is a PI3K subunit exclusively expressed in leukocytes. Patients heterozygous for this mutation are now said to have “activated PI3Kδ syndrome,” or APDS, of which ~200 patients have been described to date (88). Activated PI3Kδ syndrome is characterized by impaired T- and B-cell development and function, autoimmunity, and lymphoproliferation. One of the major downstream effectors of PI3K is mTOR, which regulates cell growth and survival and is critical for T_H1_ and T_FH_ cell differentiation (89, 90). While effector cells proliferate, naïve, and central memory T-cell subsets remain metabolically quiescent, likely contributing to autoimmunity, lymphoproliferation, and immunodeficiency seen in this syndrome (91). As is the case with STAT3 GOF mutations, both B cell-intrinsic and -extrinsic effects have been described as PI3Kδ is expressed in B cells and other leukocytes alike.

Nuclear factor kappa-light-chain-enhancer of activated B cells (NF-κB) is a family of transcription factors that are crucial for B-cell maturation, survival, differentiation, class switching, as well as self-tolerance (92). It is also a fundamental transcription factor for cytokine production by innate immune cells as well as other vital cell signaling pathways that expand beyond the immune system (93). NF-κB1 and NF-κB2 deficiencies were first described in patients of CVID affected families who were found to carry autosomal dominant mutations in *NFKB1* and *NFKB2* genes, respectively (94, 95). Mutations affecting the inducible T-cell co-stimulator (ICOS) are closely related to NF-κB deficiencies since NF-κB are activated by ICOS receptors. Because ICOS activation is essential for terminal B cell differentiation and immune tolerance (96) both ICOS and NF-κB deficiencies result in CVID-like immunodeficiency syndromes and autoimmunity (77). *NFKB1* mutation has also been linked with cytokine dysregulation, namely the elevation of type 1 cytokines that mirrors the immune profile characterizing the broader population of genetically-undefined CVID with non-infectious complications (97). Some with *NFKB1* mutations have antibody deficiency without associated non-infectious complications (98). *NFKB2* mutations lead to autoimmunity affecting the skin, hair and nails, such as alopecia and trachyonychia, and less frequently autoimmune cytopenias, and are characterized by pituitary hormone deficiencies (99) (Table 1).

**Table 1:** Monogenic defects associated with autoimmunity and CVID.

**Genetic defect**	**Protein**	**Immune dysregulation**	**Targeted treatment**	**Non-targeted treatment**
*TNFRSF13B* mutation	TACI defect	Variable phenotype. Single mutation considered risk factor for CVID; also found in asymptomatic individuals		Steroids, high dose immunoglobulin, rituximab, thrombopoeitin receptor agonist
*TNFRSF13C* mutation	BAFF-R defect			
1-3 *ICOS* LOF	ICOS deficiency	Autoimmune enteropathy, cytopenias, rheumatic disease		
*NFKB1* LOF	NF-κB1 deficiency	Autoimmune cytopenias and enteropathy, lymphoproliferation, lymphoma		
*NFKB2* LOF	NF-κB2 deficiency	Pituitary hormone deficiencies, autoimmune disease affecting skin, hair and nails		
1-4 *LRBA* LOF	LRBA deficiency	Severe early-onset autoimmune disease (including autoimmune cytopenias, IBD, type 1 diabetes), lymphoproliferation, atopy (food allergy, dermatitis, urticaria)	Abatacept	
*CTLA4* LOF	CTLA-4 deficiency			
1-4 *PIK3CD* GOF	PI3Kδ hyperactivity	Autoimmune cytopenias, primary sclerosing cholangitis, IBD, lymphoproliferation, lymphoma	Rapamycin; Leniolisib^*^	
1-4 *STAT3* GOF	STAT3 hyperactivity	Early onset endocrine autoimmunity (type 1 diabetes, hypothyroidism), autoimmune cytopenias, lymphoproliferation, interstitial lung disease	Tocilizumab; Jakinibs	

* *Currently in phase 3 clinical trial*.

## Microbiome in CVID and Autoimmunity

As noted above, monogenic mutations represent a minority of CVID patients with CVID. The majority of cases may be polygenic, or perhaps better defined as simply multifactorial, with environmental, epigenetic, or other factors contributing. Over the past decade, the microbiome has been implicated in the manifestation of immune dysregulation (100), and has been explored in the pathogenesis of CVID complications (46).

It has been hypothesized that impaired immunity results in increased microbial translocation across the gut barrier. This in turn drives persistent systemic immune activation leading to the disruption of the immune homeostasis (46, 101, 102). While regular immune sampling and microbial translocation occur in healthy individuals (103), the increased frequency occurring in CVID patients with diminished barrier function may lead to both local and systemic inflammation and immune dysregulation. Lipopolysaccharides (LPS) and soluble CD14 and IL-2 are used as markers for endotoxemia since their presence in systemic circulation is indicative of increased gut microbial translocation (46). CVID patients have higher levels of LPS and soluble CD14 and IL-2 compared to healthy controls, and CVID patients with autoimmune complications have higher levels compared to CVID without complications (102).

Low IgA levels are thought to play an important role in microbial dysbiosis seen in CVID since IgA is directly associated with the gastrointestinal tract immunity. Patients with isolated IgA deficiency have been found to have lower frequencies of T_R_ cells, especially in those patients with IgA deficiency and autoimmune disease (104). They have also been found to have reduced gut microbial diversity (105). Separately, a feedback loop has been described whereby healthy microbiota stimulate T_R_ cells and leads to the formation of germinal centers and IgA, which in turn maintains a healthy microbiome (106). The disruption of this feedback loop by low IgA levels and an unhealthy microbiome leads to reduction in T_R_ cells and subsequent immune dysregulation. Importantly, systemic IgG responses may be significant in preventing inflammation in those with IgA deficiency as a consequence of microbial dysbiosis (107). Such protective IgG responses may be significantly impaired in some CVID patients, thus predisposing to inflammation and, potentially, autoimmune disease.

Similarly to IgA deficient patients, CVID patients' gut microbial diversity was found to be reduced compared to healthy controls, with again, higher levels of soluble IL-2 and LPS detected in CVID patients with inflammatory and autoimmune complications (102). They also found an inverse correlation between T cell activation and gut microbial diversity, again suggesting that those with reduced microbial diversity have higher levels of T cell activation, and thus autoimmunity. While these findings offer insight into some drivers of immune dysregulation, further work is needed to unravel the specific mechanisms by which the microbiome affects the immune system, and how it is altered in patients with CVID, to best understand how it should be modulated or otherwise harnessed to treat complications of this immune deficiency.

## Treatment of Autoimmunity in CVID

Classically, autoimmunity in CVID has been treated with broad immunosuppressive agents, including corticosteroids, methotrexate, and azathioprine among others, which place already immunodeficient patients at an even higher risk of infections. For genetically undefined CVID, the use of rituximab for autoimmune cytopenias in CVID is one of the most efficacious and safe treatments. Its use was first documented in 2004 (108), and its efficacy and safety have been well-established, especially for ITP (109). While ritixumab's efficacy with autoimmune cytopenias may be in part due to its B-cell depleting properties resulting in depletion of autoantibodies, it is thought that its success in CVID patients is also partially due to its effect on T cells (110), again highlighting the importance of T cell abnormalities in CVID. There is the documented potential risk of persistent B-cell lymphopenia after treatment with rituximab (111), but this risk is offset by the ongoing use of immunoglobulin replacement therapy. Other therapies include thrombopoietin-receptor agonists, such as romiplostim and eltrombopag which were approved by the FDA in 2008 for the treatment of cirrhosis-associated thrombocytopenia, have shown success in the treatment of thrombocytopenia in CVID and other immunodeficiencies (112, 113).

In recent years, thanks to the recent molecular and genetic findings in CVID, more targeted approaches have led to improved results through precision medicine therapy. In CTLA-4 deficiency, corticosteroids have been the most consistently used immunosuppressive agents for autoimmunity, while other steroid-sparing agents (such as mycophenolate mofetil, cyclosporine, rituximab, anti-TNF drugs) have had mixed results (114). Abatacept, a CTLA-4 immunoglobulin fusion protein, considered as CTLA-4 replacement precision therapy for these patients, has been used to treat autoimmune manifestations and shown promising results. Ten patients treated with abatacept, showed either complete resolution or partial response with regards to stabilization of their cytopenia and improvement in gastrointestinal symptoms, with no reports of adverse outcomes (114, 115). Since LRBA deficiency is related to CTLA-4 deficiency as described above, it is no surprise that abatacept has also shown similar results in LRBA-deficient patients (84). These precision therapy approaches exemplify the potential of harnessing genomics and fundamental biology to improve care of patients with PID.

STAT3 activation occurs downstream of IL-6 signaling, a cytokine implicated in autoimmune disease, such as rheumatoid arthritis (86). Thus, upon discovery of STAT3 GOF mutations, IL-6 emerged as a potential target for treatment. Tocilizumab, an IL-6 receptor antagonist, was trialed successfully in 2015 in a patient with STAT3 GOF mutation who had failed other treatments; their T_H17_ cells which were elevated pre-treatment, similarly to what has been observed in other patients, normalized after treatment with tocilizumab (85). More recently, jakinibs, inhibitors of Janus kinases (JAKs) which are also involved in the activation cascade of STAT3, have been used adjunctly with tocilizumab in six patients and have yielded more sustained results (116). The authors of this latest study suggest that the combination of IL-6 blockade and a jakinib may be the most effective treatment strategy for patients with STAT3 GOF mutations (116).

Targeted therapy in PI3Kδ mutations have focused on the mTOR pathway and its inhibitor, rapamycin. A cohort of 26 APDS patients from the ESID registry were treated with rapamycin which showed excellent effects on the lymphoproliferative aspect of the disease, but less promising results on autoimmune cytopenias and enteropathy (117). Directed targeted inhibition of PI3Kδ is being explored with the use of leniolisib: early results of the clinical trial published in 2017 showed improvement in lymphoproliferation as well as cytopenias (118). Nemiralisib, an inhaled PI3Kδ inhibitor, has thus far shown safety and tolerability in healthy, asthmatic, and COPD patients (91) but no data has been published on APDS patients as of the publication of this review. The field of CVID has grown by leaps and bounds in recent years, coupling genomic studies with precision therapeutic approaches that have significantly improved both the efficacy and tolerability of immunosuppressive treatment for non-infectious complications.

## Conclusion

Autoimmunity in CVID is profoundly shaped by the nature of immune dysregulation that accompanies the immune deficiency in these patients. For many years CVID patients with autoimmunity have been set apart from other CVID patients on the basis of immunophenotypic characteristics, but recent advances in our understanding of genetic defects associated with CVID have shed light on the underlying pathophysiology of autoimmunity. This improved understanding has inspired treatment of autoimmunity with targeted therapies in patients who would otherwise be subjected to broad immunosuppression. Some monogenic defects have now been listed as separate immunodeficiency syndromes, such as APDS. Importantly, many of these monogenic defects have variable clinical presentation, attributed to incomplete penetrance or variable expressivity. Additional factors, such as microbial dysbiosis, may also contribute to the pathophysiology of the disease, leading to greater heterogeneity. Future research that focuses on the immune dysregulation caused by the alteration of the gut microbiota may lead to a completely new line of therapies for these patients, such as probiotics, fecal transplantation, or even dietary recommendations. Examination of large CVID cohorts in non-Western countries may shed further light on these alternative mechanisms that may shape disease manifestations, especially given profound dietary differences and possible changes in gut microbiota between globally diverse populations. Ethnic, racial, gender, and socioeconomic factors are likely to be important to explore within Western countries. As we continue to understand the mechanisms underlying the multifactorial physiology that underlies CVID disorders, we will move closer to elucidating the fundamental immune changes that can be targeted with precision therapies to optimize disease management.

## Author Contributions

JG wrote the review with support from PM.

### Conflict of Interest

The authors declare that the research was conducted in the absence of any commercial or financial relationships that could be construed as a potential conflict of interest.
